# 3-Fluoro­benzoic acid–4-acetyl­pyridine (1/1) at 100 K

**DOI:** 10.1107/S1600536809002396

**Published:** 2009-01-23

**Authors:** Gavin A. Craig, Lynne H. Thomas, Martin S. Adam, Angela Ballantyne, Andrew Cairns, Stephen C. Cairns, Gary Copeland, Clifford Harris, Eve McCalmont, Robert McTaggart, Alan R. G. Martin, Sarah Palmer, Jenna Quail, Harriet Saxby, Duncan J. Sneddon, Graeme Stewart, Neil Thomson, Alex Whyte, Chick C. Wilson, Andrew Parkin

**Affiliations:** a WestCHEM, Department of Chemistry, University of Glasgow, University Avenue, Glasgow G12 8QQ, Scotland; b Department of Chemistry, University of Glasgow, University Avenue, Glasgow G12 8QQ, Scotland

## Abstract

In the title compound, C_7_H_5_FO_2_·C_7_H_7_NO, a moderate-strength hydrogen bond is formed between the carboxyl group of one mol­ecule and the pyridine N atom of the other. The benzoic acid mol­ecule is observed to be disordered over two positions with the second orientation only 4% occupied. This disorder is also reflected in the presence of diffuse scattering in the diffraction pattern.

## Related literature

For the structure of pure *m*-fluoro­benzoic acid, see: Taga *et al.* (1985 ▶). For standard bond-length data, see: Allen *et al.* (1992 ▶).
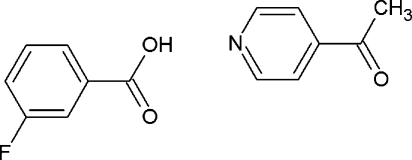


## Experimental

### 

#### Crystal data

C_7_H_5_FO_2_·C_7_H_7_NO
*M* *_r_* = 261.25
Monoclinic, 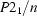

*a* = 10.0498 (11) Å
*b* = 10.5779 (8) Å
*c* = 11.5045 (8) Å
β = 92.026 (4)°
*V* = 1222.23 (18) Å^3^
*Z* = 4
Mo *K*α radiation
μ = 0.11 mm^−1^
*T* = 100 (2) K
0.3 × 0.25 × 0.2 mm

#### Data collection

Rigaku R-AXIS RAPID IP image-plate diffractometer
Absorption correction: none
15130 measured reflections
2787 independent reflections
1888 reflections with *I* > 2σ(*I*)
*R*_int_ = 0.031

#### Refinement

*R*[*F* ^2^ > 2σ(*F* ^2^)] = 0.035
*wR*(*F* ^2^) = 0.099
*S* = 1.05
2787 reflections
220 parameters
1 restraint
H atoms treated by a mixture of independent and constrained refinement
Δρ_max_ = 0.23 e Å^−3^
Δρ_min_ = −0.21 e Å^−3^

### 

Data collection: *CrystalClear* (Rigaku, 2008 ▶); cell refinement: *CrystalClear*; data reduction: *CrystalClear*; program(s) used to solve structure: *SHELXS97* (Sheldrick, 2008 ▶); program(s) used to refine structure: *SHELXL97* (Sheldrick, 2008 ▶); molecular graphics: *ORTEP-3* (Farrugia, 1997 ▶) and *Mercury* (Macrae *et al.*, 2006 ▶); software used to prepare material for publication: *WinGX* (Farrugia, 1999 ▶).

## Supplementary Material

Crystal structure: contains datablocks global, I. DOI: 10.1107/S1600536809002396/lh2755sup1.cif

Structure factors: contains datablocks I. DOI: 10.1107/S1600536809002396/lh2755Isup2.hkl

Additional supplementary materials:  crystallographic information; 3D view; checkCIF report

## Figures and Tables

**Table 1:** Hydrogen-bond geometry (Å, °)

*D*—H⋯*A*	*D*—H	H⋯*A*	*D*⋯*A*	*D*—H⋯*A*
O9—H9⋯N11	0.97 (2)	1.68 (2)	2.6428 (14)	176.3 (18)
C19—H19*C*⋯O10^i^	0.96 (2)	2.57 (2)	3.385 (2)	142.5 (14)
C13—H13⋯F1^ii^	0.98 (2)	2.63 (2)	3.3870 (16)	134.3 (12)

Symmetry codes: (i) 
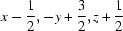
; (ii) 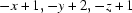.

**Fig. 1.**
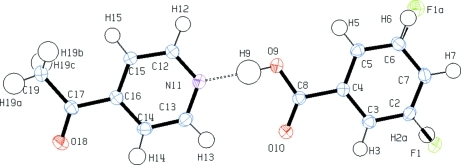
The title complex with displacement ellipsoids drawn at the 50% probability level. The minor disordered component is represented by F1a and H2a. The intermolecular hydrogen bond is indicated by a dashed line.

**Fig. 2.**
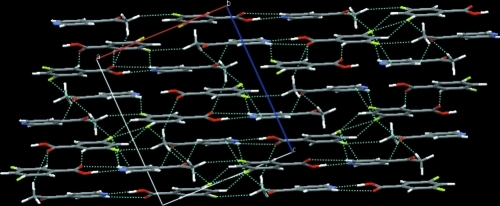
Packing figure viewed along the b axis. The tilted layer structure can be clearly seen and close intermolecular C—H···F and C—H···O distances are represented by dotted lines.

**Fig. 3.**
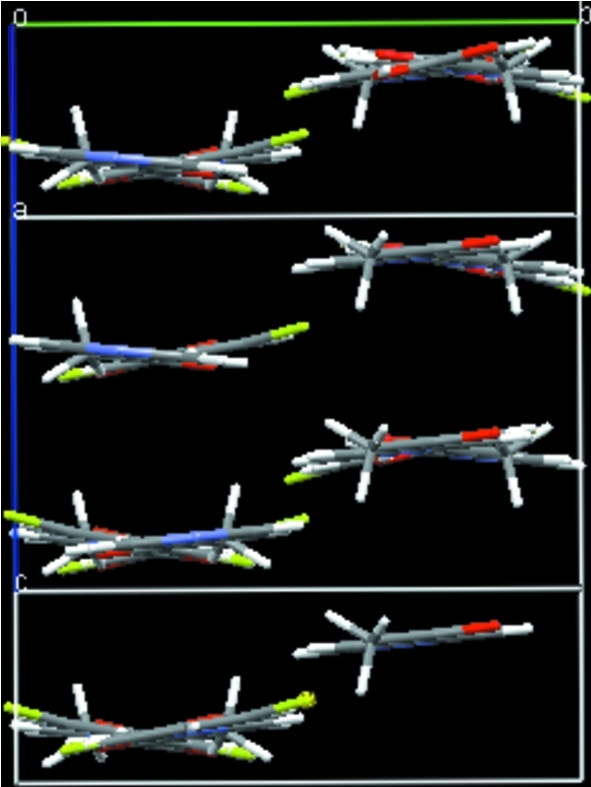
Packing view to illustrate the offset from planarity of the various units. This figure shows exactly the same molecules as in Fig. 2, but rotated by 90°.

## supplementary crystallographic information

### Comment

The structure of a molecular complex of 3-fluorobenzoic acid with 4-acetylpyridine (C_7_H_5_O_2_F C_7_H_7_NO) at 100 K is reported (Fig. 1). The molecular geometry of the 4-acetylpyridine is generally unremarkable. However the F atom in the 3-fluorobenzoic acid molecule is seen to be disordered over two positions in a 96:4% ratio. The majority component is found to lie on the same side of the molecule as the carbonyl C═O and this is consistent with the reported crystal structure of the pure material (Taga *et al.*, 1985). The minority component was identified using a Fourier difference map which shows a peak height of greater than 1 electron bonded to C6, at a distance longer than characteristic for a C—H bond. The inclusion of a disorder model even at the 4% level improves the model significantly. Diffuse scattering was also observed in the diffraction images supporting the presence of disorder in this material. The minor component F atom was modelled isotropically and with constraints on the C—F distance. The thermal ellipsoids of both the carboxylic acid group and the methyl-keto group are slightly larger than those of their corresponding aromatic rings, indicating the possible presence of a small amount of libration in these groups.

A moderate strength hydrogen bond [O···N = 2.6428 (14) Å] is formed between the carboxylic acid group and the pyridine N atom. There is no indication of disorder of the carboxylic H atom at this temperature although the H atom isotropic thermal parameter is large as is often observed in the presence of a hydrogen bond. The two molecules lie almost exactly co-planar with each other.

These dimers are packed in an offset planar arrangement, as shown in Figs. 2 and 3. All the molecules are approximately co-planar with the (103) plane. The reason that this offset occurs may be due to the optimization of two close contacts from the methyl group. These contacts comprise a C—H···O interaction between the methyl group of the 4-acetylpyridine and the C═O of the carboxylic acid between planes, which induces an attractive tilt upwards in the acetylpyridine towards this acid molecule. Equally, the C—H···F interaction within the plane causes an attractive tilt in the adjacent molecule, giving rise to this offset packing arrangement.

### Experimental

Crystals of the title material were grown by slow evaporation of solvent from a 1:1 solution of the two component molecules in ethanol.

### Refinement

All non-H atoms were refined anisotropically except that of the disordered F atom where the minor component was left isotropic. The C—F distances for the minor and major components were constrained to be similar. All H atoms were identified in the difference map, and were allowed to refine isotropically with the exception of the disordered positions where they were fixed geometrically and refined as riding groups. The proportion of disorder was obtained by identifying the value which gave the lowest *R*-factor.

### Crystal data

**Table:**

C_7_H_5_FO_2_·C_7_H_7_NO	*F*(000) = 544
*M**_r_* = 261.25	*D*_x_ = 1.42 Mg m^−^^3^
Monoclinic, *P*2_1_/*n*	Mo *K*α radiation, λ = 0.71073 Å
Hall symbol: -P 2yn	Cell parameters from 10265 reflections
*a* = 10.0498 (11) Å	θ = 3–28°
*b* = 10.5779 (8) Å	µ = 0.11 mm^−^^1^
*c* = 11.5045 (8) Å	*T* = 100 K
β = 92.026 (4)°	Block, colourless
*V* = 1222.23 (18) Å^3^	0.3 × 0.25 × 0.2 mm
*Z* = 4	

### Data collection

**Table:**

Rigaku R-AXIS RAPID IP image-plate diffractometer	*R*_int_ = 0.031
ω scans	θ_max_ = 27.5°, θ_min_ = 3.3°
15130 measured reflections	*h* = −13→13
2787 independent reflections	*k* = −13→11
1888 reflections with *I* > 2σ(*I*)	*l* = −13→14

### Refinement

**Table:**

Refinement on *F*^2^	1 restraint
Least-squares matrix: full	H atoms treated by a mixture of independent and constrained refinement
*R*[*F*^2^ > 2σ(*F*^2^)] = 0.035	*w* = 1/[σ^2^(*F*_o_^2^) + (0.0573*P*)^2^] where *P* = (*F*_o_^2^ + 2*F*_c_^2^)/3
*wR*(*F*^2^) = 0.099	(Δ/σ)_max_ = 0.001
*S* = 1.05	Δρ_max_ = 0.23 e Å^−^^3^
2787 reflections	Δρ_min_ = −0.21 e Å^−^^3^
220 parameters	

### Special details

**Table:**

Geometry. All e.s.d.'s (except the e.s.d. in the dihedral angle between two l.s. planes) are estimated using the full covariance matrix. The cell e.s.d.'s are taken into account individually in the estimation of e.s.d.'s in distances, angles and torsion angles; correlations between e.s.d.'s in cell parameters are only used when they are defined by crystal symmetry. An approximate (isotropic) treatment of cell e.s.d.'s is used for estimating e.s.d.'s involving l.s. planes.

### Fractional atomic coordinates and isotropic or equivalent isotropic displacement parameters (Å^2^)

**Table:**

	*x*	*y*	*z*	*U*_iso_*/*U*_eq_	Occ. (<1)
F1	0.86852 (8)	0.91451 (8)	0.42397 (7)	0.0336 (2)	0.96
C2	0.80192 (13)	0.81276 (12)	0.46507 (11)	0.0243 (3)	0.96
C2A	0.80192 (13)	0.81276 (12)	0.46507 (11)	0.0243 (3)	0.04
H2A	0.8481	0.8820	0.4373	0.029*	0.04
C3	0.67564 (13)	0.83201 (12)	0.50485 (11)	0.0220 (3)	
H3	0.6362 (14)	0.9146 (14)	0.5003 (11)	0.024 (4)*	
C4	0.60718 (12)	0.72721 (12)	0.54569 (10)	0.0203 (3)	
C5	0.66565 (13)	0.60822 (12)	0.54596 (10)	0.0229 (3)	
H5	0.6176 (15)	0.5344 (14)	0.5736 (12)	0.029 (4)*	
C6	0.79390 (13)	0.59376 (13)	0.50555 (11)	0.0251 (3)	0.96
H6	0.8334	0.5142	0.5063	0.030*	0.96
C6A	0.79390 (13)	0.59376 (13)	0.50555 (11)	0.0251 (3)	0.04
F1A	0.8666 (19)	0.4867 (15)	0.5104 (17)	0.033 (4)*	0.04
C7	0.86291 (14)	0.69651 (13)	0.46440 (11)	0.0256 (3)	
H7	0.9528 (16)	0.6854 (14)	0.4351 (12)	0.027 (4)*	
C8	0.46965 (13)	0.74749 (11)	0.58875 (10)	0.0211 (3)	
O9	0.41636 (10)	0.64448 (9)	0.63191 (8)	0.0299 (2)	
H9	0.325 (2)	0.6594 (19)	0.6527 (17)	0.068 (6)*	
O10	0.41357 (9)	0.84903 (9)	0.58340 (8)	0.0287 (2)	
N11	0.16961 (11)	0.68081 (10)	0.69676 (9)	0.0235 (3)	
C12	0.09586 (13)	0.58338 (13)	0.73125 (11)	0.0235 (3)	
H12	0.1365 (15)	0.4988 (14)	0.7270 (11)	0.026 (4)*	
C13	0.11730 (14)	0.79700 (13)	0.70272 (11)	0.0254 (3)	
H13	0.1748 (15)	0.8649 (15)	0.6758 (12)	0.030 (4)*	
C14	−0.00901 (13)	0.81965 (13)	0.74237 (11)	0.0240 (3)	
H14	−0.0396 (15)	0.9059 (16)	0.7441 (13)	0.033 (4)*	
C15	−0.03229 (13)	0.59762 (12)	0.77072 (10)	0.0213 (3)	
H15	−0.0813 (14)	0.5233 (14)	0.7917 (12)	0.026 (4)*	
C16	−0.08659 (12)	0.71782 (12)	0.77594 (10)	0.0208 (3)	
C17	−0.22611 (13)	0.74106 (12)	0.81607 (10)	0.0222 (3)	
O18	−0.26975 (10)	0.84818 (9)	0.81733 (9)	0.0327 (3)	
C19	−0.30654 (14)	0.63097 (14)	0.85455 (13)	0.0282 (3)	
H19A	−0.399 (2)	0.6527 (17)	0.8522 (15)	0.049 (5)*	
H19B	−0.2946 (17)	0.5564 (17)	0.8057 (14)	0.045 (5)*	
H19C	−0.2765 (17)	0.6106 (17)	0.9328 (15)	0.050 (5)*	

### Atomic displacement parameters (Å^2^)

**Table:**

	*U*^11^	*U*^22^	*U*^33^	*U*^12^	*U*^13^	*U*^23^
F1	0.0277 (5)	0.0260 (5)	0.0480 (5)	−0.0030 (3)	0.0144 (4)	0.0045 (4)
C2	0.0239 (7)	0.0238 (7)	0.0255 (7)	−0.0041 (5)	0.0044 (5)	0.0021 (5)
C2A	0.0239 (7)	0.0238 (7)	0.0255 (7)	−0.0041 (5)	0.0044 (5)	0.0021 (5)
C3	0.0236 (7)	0.0210 (7)	0.0215 (6)	0.0006 (5)	0.0014 (5)	−0.0009 (5)
C4	0.0203 (6)	0.0220 (7)	0.0186 (6)	−0.0006 (5)	0.0003 (5)	−0.0013 (5)
C5	0.0258 (7)	0.0223 (7)	0.0207 (6)	−0.0009 (5)	0.0013 (5)	0.0016 (5)
C6	0.0265 (7)	0.0232 (7)	0.0257 (6)	0.0045 (5)	0.0031 (5)	−0.0007 (5)
C6A	0.0265 (7)	0.0232 (7)	0.0257 (6)	0.0045 (5)	0.0031 (5)	−0.0007 (5)
C7	0.0215 (7)	0.0299 (7)	0.0255 (7)	0.0032 (5)	0.0047 (5)	0.0010 (5)
C8	0.0203 (6)	0.0209 (7)	0.0221 (6)	−0.0018 (5)	0.0008 (5)	−0.0012 (5)
O9	0.0223 (5)	0.0251 (5)	0.0428 (6)	−0.0002 (4)	0.0101 (4)	0.0039 (4)
O10	0.0251 (5)	0.0224 (5)	0.0391 (5)	0.0015 (4)	0.0081 (4)	0.0013 (4)
N11	0.0207 (5)	0.0241 (6)	0.0259 (6)	−0.0014 (4)	0.0029 (4)	0.0015 (4)
C12	0.0235 (7)	0.0224 (7)	0.0247 (7)	0.0011 (5)	0.0029 (5)	−0.0003 (5)
C13	0.0254 (7)	0.0230 (7)	0.0278 (7)	−0.0033 (6)	0.0015 (5)	0.0021 (5)
C14	0.0256 (7)	0.0189 (7)	0.0275 (7)	0.0002 (5)	−0.0006 (5)	−0.0003 (5)
C15	0.0220 (6)	0.0189 (7)	0.0231 (6)	−0.0010 (5)	0.0018 (5)	0.0009 (5)
C16	0.0197 (6)	0.0227 (7)	0.0199 (6)	0.0000 (5)	−0.0018 (5)	−0.0021 (5)
C17	0.0211 (7)	0.0225 (7)	0.0228 (6)	0.0018 (5)	−0.0013 (5)	−0.0037 (5)
O18	0.0270 (5)	0.0236 (5)	0.0477 (6)	0.0054 (4)	0.0055 (4)	−0.0027 (4)
C19	0.0205 (7)	0.0279 (8)	0.0364 (8)	0.0003 (6)	0.0048 (6)	−0.0001 (6)

### Geometric parameters (Å, °)

**Table:**

F1—C2	1.3611 (15)	N11—C13	1.3394 (17)
C2—C7	1.3740 (19)	C12—C15	1.3888 (18)
C2—C3	1.3794 (18)	C12—H12	0.985 (15)
C3—C4	1.3949 (18)	C13—C14	1.3848 (19)
C3—H3	0.960 (15)	C13—H13	0.979 (16)
C4—C5	1.3890 (18)	C14—C16	1.3923 (18)
C4—C8	1.5001 (18)	C14—H14	0.963 (16)
C5—C6	1.3940 (18)	C15—C16	1.3858 (18)
C5—H5	0.978 (15)	C15—H15	0.963 (15)
C6—C7	1.3819 (19)	C16—C17	1.5118 (17)
C6—H6	0.9300	C17—O18	1.2153 (15)
C7—H7	0.982 (16)	C17—C19	1.4935 (19)
C8—O10	1.2135 (15)	C19—H19A	0.955 (19)
C8—O9	1.3189 (15)	C19—H19B	0.978 (17)
O9—H9	0.97 (2)	C19—H19C	0.964 (18)
N11—C12	1.3378 (17)		
			
F1—C2—C7	118.74 (11)	N11—C12—H12	116.7 (9)
F1—C2—C3	117.91 (12)	C15—C12—H12	120.4 (9)
C7—C2—C3	123.35 (12)	N11—C13—C14	122.79 (12)
C2—C3—C4	117.71 (12)	N11—C13—H13	114.8 (9)
C2—C3—H3	119.9 (8)	C14—C13—H13	122.4 (9)
C4—C3—H3	122.3 (8)	C13—C14—C16	119.10 (12)
C5—C4—C3	120.48 (12)	C13—C14—H14	117.9 (9)
C5—C4—C8	121.57 (11)	C16—C14—H14	123.0 (9)
C3—C4—C8	117.95 (11)	C16—C15—C12	119.00 (12)
C4—C5—C6	119.65 (12)	C16—C15—H15	122.1 (9)
C4—C5—H5	120.7 (9)	C12—C15—H15	118.8 (9)
C6—C5—H5	119.6 (9)	C15—C16—C14	118.20 (12)
C7—C6—C5	120.60 (13)	C15—C16—C17	122.23 (11)
C7—C6—H6	119.7	C14—C16—C17	119.57 (12)
C5—C6—H6	119.7	O18—C17—C19	121.61 (12)
C2—C7—C6	118.20 (12)	O18—C17—C16	119.62 (12)
C2—C7—H7	121.6 (9)	C19—C17—C16	118.77 (11)
C6—C7—H7	120.2 (9)	C17—C19—H19A	109.9 (11)
O10—C8—O9	123.80 (12)	C17—C19—H19B	112.3 (10)
O10—C8—C4	122.79 (11)	H19A—C19—H19B	108.4 (14)
O9—C8—C4	113.41 (11)	C17—C19—H19C	107.2 (11)
C8—O9—H9	111.0 (12)	H19A—C19—H19C	110.6 (14)
C12—N11—C13	117.97 (11)	H19B—C19—H19C	108.4 (14)
N11—C12—C15	122.91 (12)		
			
F1—C2—C3—C4	179.30 (11)	C3—C4—C8—O9	−176.16 (11)
C7—C2—C3—C4	−0.3 (2)	C13—N11—C12—C15	1.11 (19)
C2—C3—C4—C5	0.14 (18)	C12—N11—C13—C14	−0.25 (19)
C2—C3—C4—C8	−179.83 (11)	N11—C13—C14—C16	−1.1 (2)
C3—C4—C5—C6	0.28 (18)	N11—C12—C15—C16	−0.61 (19)
C8—C4—C5—C6	−179.75 (11)	C12—C15—C16—C14	−0.72 (18)
C4—C5—C6—C7	−0.5 (2)	C12—C15—C16—C17	179.02 (11)
F1—C2—C7—C6	−179.53 (11)	C13—C14—C16—C15	1.51 (19)
C3—C2—C7—C6	0.1 (2)	C13—C14—C16—C17	−178.24 (11)
C5—C6—C7—C2	0.3 (2)	C15—C16—C17—O18	−178.87 (11)
C5—C4—C8—O10	−175.77 (11)	C14—C16—C17—O18	0.87 (18)
C3—C4—C8—O10	4.20 (19)	C15—C16—C17—C19	1.77 (18)
C5—C4—C8—O9	3.87 (17)	C14—C16—C17—C19	−178.49 (12)

### Hydrogen-bond geometry (Å, °)

**Table:**

*D*—H···*A*	*D*—H	H···*A*	*D*···*A*	*D*—H···*A*
O9—H9···N11	0.97 (2)	1.68 (2)	2.6428 (14)	176.3 (18)
C19—H19C···O10^i^	0.96 (2)	2.57 (2)	3.385 (2)	142.5 (14)
C13—H13···F1^ii^	0.98 (2)	2.63 (2)	3.3870 (16)	134.3 (12)

Symmetry codes: (i) *x*−1/2, −*y*+3/2, *z*+1/2; (ii) −*x*+1, −*y*+2, −*z*+1.
